# Clinical application of FIGO 2023 staging system of endometrial cancer in a Chinese cohort

**DOI:** 10.1186/s12885-024-12633-8

**Published:** 2024-07-18

**Authors:** Changmin Yu, Xinhui Yuan, Qianlan Yao, Yuyin Xu, Xiaoyan Zhou, Xin Hu, Huijuan Yang, Huaying Wang, Xiaoli Zhu, Yulan Ren

**Affiliations:** 1 Department of Gynecologic Oncology, Fudan University Shanghai Cancer Center, Shanghai, 200032 China; 2 Department of Oncology, Shanghai Medical College, Fudan University, Shanghai, 200032 China; 3 Department of Pathology, Fudan University Shanghai Cancer Center, Shanghai, 200032 China; 4 Institute of Pathology, Fudan University, Shanghai, 200032 China; 5 Precision Cancer Medicine Center, Fudan University Shanghai Cancer Center, Shanghai, China

**Keywords:** Endometrial cancer, Staging, Molecular classification, Prognosis, Survival

## Abstract

**Objective:**

The International Federation of Gynecology and Obstetrics (FIGO) 2023 staging system for endometrial cancer (EC) was released with incorporating histology, lympho-vascular space invasion, and molecular classification together. Our objective is to further explore the clinical utility and prognostic significance of the 2023 FIGO staging system in China.

**Methods:**

A retrospective analysis was conducted for patients who received standard surgeries and underwent genetic testing using multigene next-generation sequencing (NGS) panels between December 2018 and December 2023 at Fudan University Shanghai Cancer Center, Shanghai, China. The genomic and clinical data of all patients were analyzed, and stages were determined by both the 2009 and 2023 FIGO staging systems. Kaplan–Meier estimators and Cox proportional hazards models were used for survival analysis.

**Results:**

A total of 547 patients were enrolled in the study. After the restaged by the FIGO 2023 staging system, stage shifts occurred in 147/547 (26.9%) patients. In patients with early stages in FIGO 2009 (stage I-II), 63 cases were rearranged to IAm*POLE*mut and 53 cases to IICmp53abn due to the molecular classification of *POLE*mut and p53abn. Altogether 345 cases were in stage I, 107 cases in stage II, 69 cases in stage III, and 26 cases in stage IV according to the FIGO 2023 staging criteria. For stage I diseases, the 3-year PFS rate was 92.7% and 95.3% in 2009 and 2023 FIGO staging systems, respectively. The 3-year PFS of stage II in 2023 FIGO was lower than that of FIGO 2009 (3-year PFS: 85.0% versus 90.9%), especially in substage IIC and IICmp53abn. Three cases (12%) of stage IIIA in FIGO 2009 were shifted to stage IA3 FIGO 2023, with 3-year PFS rates of 90.9% versus 100%, respectively. In NGS analysis, the most prevalent gene alterations were observed in PTEN and PIK3CA.

**Conclusion:**

The FIGO 2023 staging system was proved to be a good predictor of survival for EC patients with enhanced precision compared to FIGO 2009. Predominant stage shifts were observed in early-stage diseases. Distinct gene alterations of different subtypes may help to explore more accurate target therapies.

**Supplementary Information:**

The online version contains supplementary material available at 10.1186/s12885-024-12633-8.

## Introduction

Endometrial cancer (EC) is one of the most common gynecological cancers for women with an increasing incidence worldwide [1, 2]. It has been fifteen years since the International Federation of Gynecology and Obstetrics released the FIGO 2009 staging system for EC. In 2013, The Cancer Genome Atlas (TCGA) published a molecular classification with distinct molecular characteristics and prognostic predictions. Simplified classifications of the ProMisE and Trans-PORTEC had markedly enhanced the clinical utility of molecular classification, which include *POLE* ultramutation (*POLE*mut), mismatch repair deficiency (MMRd), p53- abnormality (p53abn), and no specific molecular profile (NSMP) [3, 4].

With the accumulated data on the prognostic significance of molecular classification and clinical characteristics, the new 2023 FIGO staging system has been released, incorporating molecular classification, lympho-vascular space invasion (LVSI), and invasive pathological type as significant prognostic indicators [5–9]. The 2023 update improves the existing staging system by providing more precise indications for prognoses and treatment of EC.

After the 2023 FIGO staging system was released, it has not been widely applied yet, especially in China. Although several retrospective studies had reported the improved prediction of prognosis with the 2023 FIGO staging system [10–12], some scholars still wondered if it was too early to incorporate evolving premature and complicated variables that lack robust supporting evidence, such as LVSI and aggressive histology [13, 14]. Thus, we conducted this retrospective study to further evaluate the clinical applicability of the FIGO2023 staging system and explore its prognostic significance of endometrial cancer.

## Materials and methods

We collected data from patients who received standard surgeries and genetic testing at Fudan University Shanghai Cancer Center (FUSCC) between December 2018 and December 2023, approved by the Institutional Review Board (IRB), which included surgical details, pathological reports, immunohistochemistry (IHC) results, postoperative therapies, and follow-up details. The inclusion and exclusion criteria are in Supplementary Fig. 1. The stage was determined by both the 2009 and 2023 FIGO staging systems. All pathological results were reviewed by at least two senior pathologists. The multi-gene next-generation sequencing (NGS) panel was utilized, including APC, AKT1, ATM, BRAF, BRCA1, BRCA2, CDH1, CHEK2, EGFR, EPCAM, ERBB2, HRAS, KIT, KRAS, MET, MLH1, MSH2, MSH6, MUTYH, NRAS, PDGFRA, PIK3CA, PMS2, POLD1, POLE, PTCH1, PTEN, SDHB, SDHC, SDHD, SMAD4, STK11, TP53, and 66 microsatellite loci, etc. (Supplementary Table 1). The NGS panel detected insertions, deletions, base substitutions, and copy number alterations of the assessed genes, as well as microsatellite instability (MSI) status. Patients were classified into POLE mutated (*POLE*mut), mismatch repair deficient (MMRd), no specific molecular profile (NSMP), or p53 abnormal (p53abn) subtypes, according to the results of NGS and MSI status as previous studies suggested [15, 16]. The molecular classification was determined by at least two senior pathologists. Sub-stratification of stages IIIC1 and IIIC2 was not evaluated due to flawed information on lymph node micro- and macro-metastasis assessments.

Statistical analyses were performed using R Studio (version 4.2.1; R Studio, http://www.R-project.org) and IBM SPSS Statistics (Version 26.0). A significance level of *p* < 0.05 was considered statistically significant. Patients’ clinicopathologic characteristics were presented, and staging shifts were depicted using both table and Sankey diagram generated with RAWGraphs (https://app.rawgraphs.io). Progression-free survival (PFS) and overall survival (OS) were assessed via Kaplan–Meier analysis, and corresponding 95% confidence intervals (CIs) were calculated using the Brookmeyer-Crowley method.

## Results

### Clinicopathologic characteristics

**Fig. 1:**
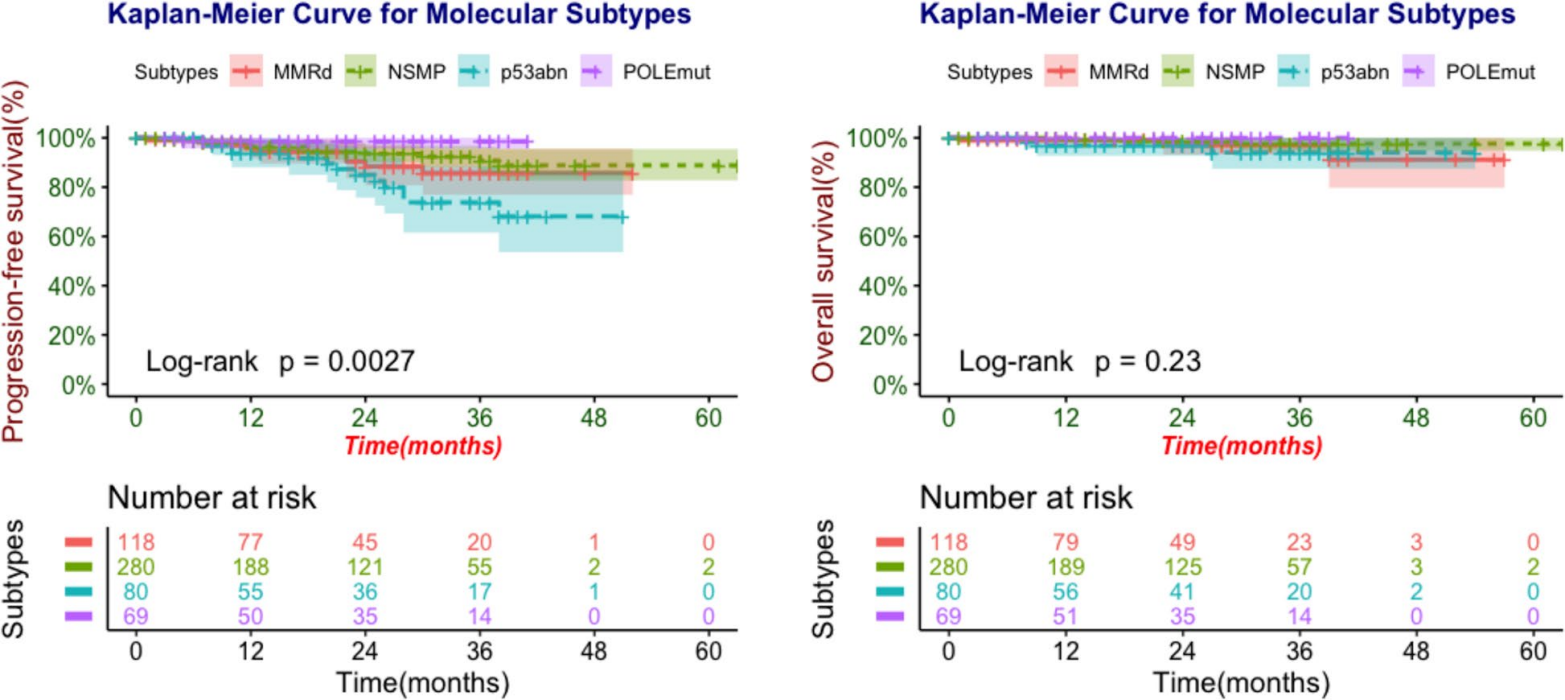
Kaplan–Meier survival curves of PFS and OS in different molecular subtypes

A total of 547 patients were enrolled in the study. According to the results of NGS, 69 *POLE*mut patients, 118 MMRd patients, 280 NSMP patients, and 80 p53abn patients were observed, respectively. PFS and OS rates of different molecular subtypes were shown in Fig. 1, in which 3-year PFS and OS of *POLE*mut patients were 98.5% [95%-confidence interval (CI) 95.6–100] and 100%, respectively. 3-year survival rates of p53abn were the lowest [3-year PFS: 73.8% (95%-CI 61.6–88.5), 3-year OS: 94.0% (95%-CI 87.4–100)]. For MMRd and NSMP subtypes, the 3-year PFS rate was 85.7% (95%-CI 76.9–95.5) versus 90.7% (95%-CI 85.6–96.2), with the 3-year OS rate of 97.2% (95%-CI 93.2–100) versus 97.6% (95%-CI 94.8–100), respectively.

The median follow-up time was 19.0 months [interquartile range (IQR): 9.0–32.0]. During the process of follow-up, 38 patients had recurrences, and 9 deaths were observed. More detailed characteristics are listed in Table 1.

**Table 1:** Patient’s characteristics (*n* = 547)

Characteristic	Number of patients (%)
**Age(year)**	54.6 (24.0–87.0)
**BMI (kg/m**^**2**^**)**	24.9 (16.4–64.4)
Underweight (< 18.5)	7 (1.3%)
Normal (18.5–22.9)	167 (30.5%)
Overweight (≥ 23.0)	373 (68.2%)
**Diabetes mellitus**	64 (11.7%)
**Hypertension**	164 (29.9%)
**Histology**	
Endometrioid	497 (90.9%)
Serous	16 (2.9%)
Clear cell	3 (0.5%)
Carcinosarcoma	7 (1.2%)
Undifferentiated	11 (20.1%)
Mixed histology	10 (18.2%)
Others	3 (0.5%)
**Grade**	
G1	211 (38.6%)
G2	213 (38.9%)
G3	76 (13.9%)
Unknown	47 (8.6%)
**Myometrial invasion**	
Confined to endometrium	72 (13.2%)
< 1/2	325 (59.4%)
≥ 1/2	150 (27.4%)
**Cervical invasion**	
Negative	67 (12.2%)
Positive	480 (87.8%)
**LVSI **^**a**^	
No LVSI	399 (72.9%)
Local LVSI	128 (23.4%)
Substantial LVSI	11 (2.0%)
Missing	9 (1.6%)
**Lymph node metastasis** ^b^	
No LN metastasis	457(83.5%)
Pelvic LN metastasis	28 (5.1%)
Paraaortic LN metastasis	2 (0.4%)
Pelvic + paraaortic LN metastasis	18 (3.3%)
Without lymphadenectomy	42 (7.7%)
**Molecular classification **^**c**^	
*POLE*mut	69 (12.6%)
MMRd	118 (21.6%)
NSMP	280 (51.2%)
P53abn	80 (14.6%)
**FIGO 2009**	
I	416 (76.1%)
II	33 (6.0%)
III	72 (13.2%)
IV	26 (4.8%)
**FIGO 2023**	
I	345 (63.1%)
II	107 (19.6%)
III	69 (12.6%)
IV	26 (4.8%)
**ER/PR status (based on IHC) **^**d**^	
ER positive	514 (93.9%)
PR positive	481 (87.9%)

^a^Lymphovascular space invasion (LVSI)

^b^With 37 cases of patients underwent sentinel lymph node biopsy; Lymph node (LN)

^c^*POLE* mutated (*POLE*mut), mismatch repair deficient (MMRd), no specific molecular profile (NSMP), or p53 abnormal (p53abn)

^d^Estrogen receptor (ER), progesterone receptor (PR), Immunohistochemistry (IHC)

### Stage shifts of patients

**Fig. 2:**
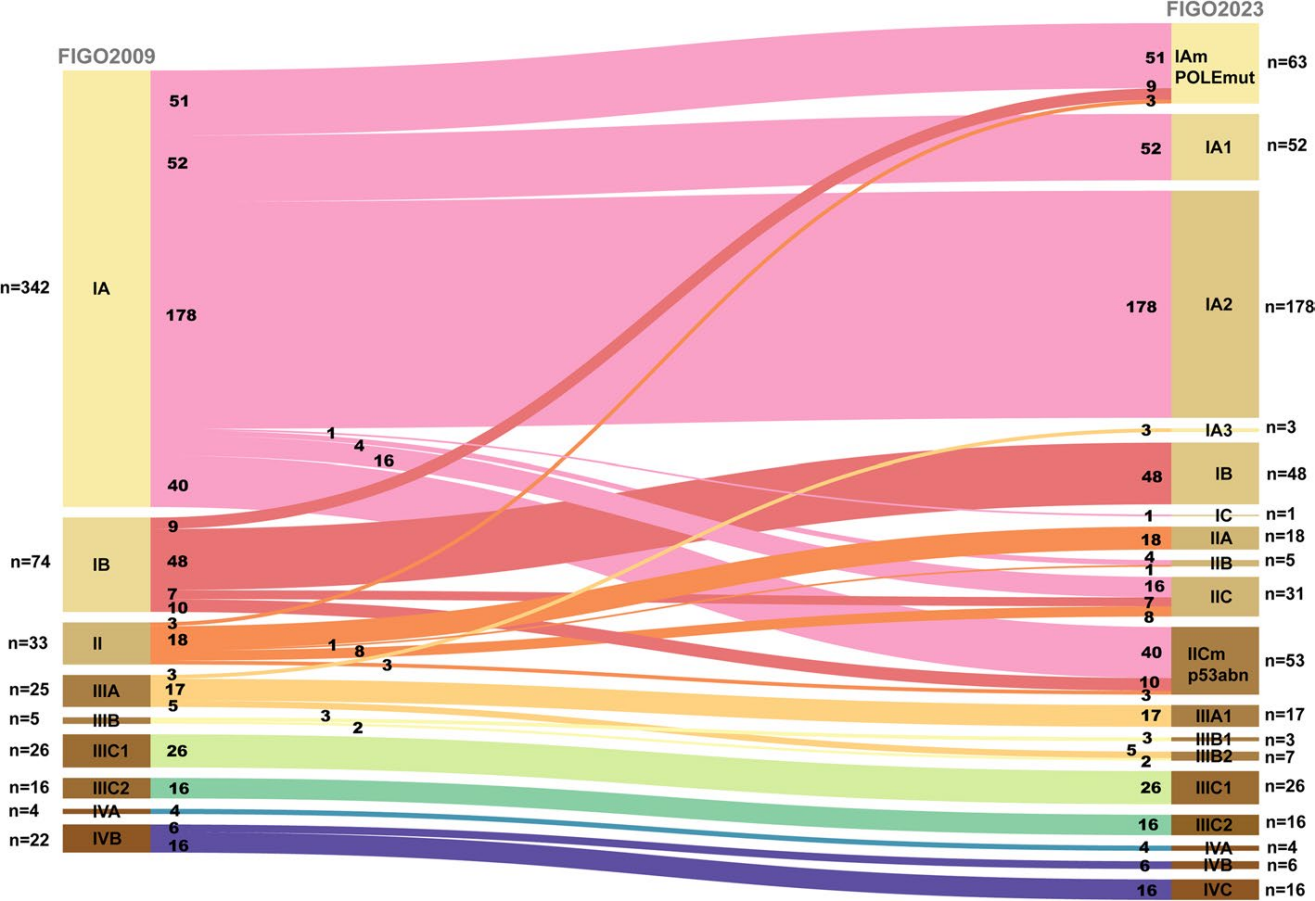
Sankey diagram of stage shifts in all patients

Among 547 patients, altogether 147 cases (26.9%) were observed with stage shifts (Table 2). Figure 2 provides the Sankey diagram of all stage shifts.

**Table 2:** FIGO 2009–2023 stage shifts in all patients

		**2009FIGO**								
		IA	IB	II	IIIA	IIIB	IIIC1	IIIC2	IVA	IVB
**2023FIGO**		*n* = 342	*n* = 74	*n* = 33	*n* = 25	*n* = 5	*n* = 26	*n* = 16	*n* = 4	*n* = 22
IAm_*POLE*mut_	*n* = 63	51	9(↓)	3(↓)	-	-	-	-	-	-
IA1	*n* = 52	52	-	-	-	-	-	-	-	-
IA2	*n* = 178	178	-	-	-	-	-	-	-	-
IA3	*n* = 3	-	-	-	3(↓)	-	-	-	-	-
IB	*n* = 48	-	48	-	-	-	-	-	-	-
IC	*n* = 1	1(↑)	-	-	-	-	-	-	-	-
IIA	*n* = 18	-	-	18	-	-	-	-	-	-
IIB	*n* = 5	4(↑)	-	1	-	-	-	-	-	-
IIC	*n* = 31	16(↑)	7(↑)	8	-	-	-	-	-	-
IICm_p53abn_	*n* = 53	40(↑)	10(↑)	3	-	-	-	-	-	-
IIIA1	*n* = 17	-	-	-	17	-	-	-	-	-
IIIB1	*n* = 3	-	-	-	-	3	-	-	-	-
IIIB2	*n* = 7	-	-	-	5(↑)	2	-	-	-	-
IIIC1	*n* = 26	-	-	-	-	-	26	-	-	-
IIIC2	*n* = 16	-	-	-	-	-	-	16	-	-
IVA	*n* = 4	-	-	-	-	-	-	-	4	-
IVB	*n* = 6	-	-	-	-	-	-	-	-	6
IVC	*n* = 16	-	-	-	-	-	-	-	-	16

In patients with early stages in 2009 FIGO (stage I-II), 63 cases (14.0%) of 2009 FIGO early-stage disease were rearranged to IAm*POLE*mut and 53 cases (11.8%) to IICmp53abn due to the molecular classification of *POLE*mut and p53abn. Stage shifts were also observed in the early stages with the incorporation of substantial lympho-vascular space invasion (LVSI) and aggressive pathological types. Four cases of 2009 FIGO stage IA converted to 2023 FIGO IIB due to substantial LVSI. Furthermore, 16 cases of stage IA (4.7%), and 7 cases of stage IB (9.5%) were reclassified as stage IIC due to aggressive histology, plus 1 case of stage IA (0.3%) converted to stage IC with aggressive histology without myometrial invasion.

In advanced-stage disease (stages III-IV), a total of 3 stage shifts were observed, which with concurrent endometrial and ovarian low-grade endometrioid cancer were restaged from IIIA under FIGO2009 to IA3 under FIGO 2023.

### Survival analysis

For stage I disease, substage IAm*POLE*mut exhibited an excellent prognosis with 3-year PFS and OS of 98.4% (95%-CI 95.3–100) and 100%, respectively. For stage II disease, the 3-year PFS in the 2023 FIGO staging system was lower than that of 2009, with 3-year PFS: 85.0% (95%-CI 75.2–96.0) versus 90.9% (95%-CI 79.5–100). The 3-year PFS for substage IIC and IICmp53abn patients was lower compared to other early-stage diseases, with 3-year PFS 72.6% (95%-CI 45.9–100) and 88.3% (95%-CI 76.6–100), respectively. For patients with stage shift of FIGO2009 stage IIIA, the 3-year PFS rates of FIGO2009 stage IIIA1 and IA3 in the 2023 FIGO staging were 87.5% (95%-CI 67.3–100) versus 100%, respectively. The prognoses of stage I-IV of FIGO 2009 and 2023 staging were detailed in Table 3.

**Table 3:** 3-year PFS and OS based on FIGO 2009 and FIGO 2023 staging system

FIGO 2009	3-year PFS rate in % (95%CI)	3-year OS rate in % (95%CI)	FIGO 2023	3-year PFS rate in % (95%CI)	3-year OS rate in % (95%CI)
**I**	**92.7(88.9–96.7)**	**99.6(98.9- /) **^**a**^	**I**	**95.3(92.1–98.6)**	**/**
IA	92.6(88.3–97.2)	/	IA1	/	/
			IA2	91.9(86.1–98.2)	95.2(86.5- /)
			IA3	/	/
			IAm*POLE*mut	98.4(95.3-/)	/
IB	92.8(84.8- /)	98.0(94.2- /)	IB	97.7(93.4-/)	/
			IC	/	/
**II**	**90.9(79.5- /)**	**94.7(85.2- /)**	**II**	**85.0(75.2–96.0)**	**96.9(92.8- /)**
			IIA	85.6(68.8- /)	92.3(78.9- /)
			IIB	/	/
			IIC	72.6(45.9- /)	94.7(85.2- /)
			IICmp53abn	88.3(76.6- /)	/
**III**	**83.3(72.0–96.4)**	**94.3(88.0- /)**	**III**	**82.8(71.1–96.3)**	**94.1(87.6- /)**
IIIA	90.9(75.4- /)	/	IIIA1	87.5(67.3- /)	/
IIIB	80.0(51.6- /)	/	IIIB1	66.7(30.0- /)	/
			IIIB2	/	/
IIIC1	91.7(77.3- /)	/	IIIC1	91.7(77.3- /)	/
IIIC2	57.7(31.6- /)	71.6(47.2- /)	IIIC2	57.7(31.6- /)	71.6(47.2- /)
**IV**	**36.4(19.1–69.3)**	**80.6(62.7- /)**	**IV**	**36.4(19.1–69.3)**	**80.6(62.7- /)**
IVA	50.0(12.5- /)	/	IVA	50.0(12.5- /)	/
IVB	33.7(16.1–70.5)	78.9(60.0- /)	IVB	20.0(3.4- /)	80.0(51.6- /)
			IVC	37.7(16.4–86.9)	80.0(58.7- /)

^a^/ refers to a survival rate of 100%

For all patients, univariate analyses showed that those with aggressive histological types (*p* < 0.0001), lymph node metastases (LNM) (*p* = 0.0054), and LVSI (*p* = 0.0015) had worse prognoses (Supplementary Fig. 2). In multivariate analyses, Grade III, LNM, non-aggressive histological subtypes, and absence of LVSI were significant prognostic factors (*p* < 0.0001), with an odds ratio (OR): 4.29, 3.09, 0.25, and 0.34, respectively. The prognoses of stage I-IV of FIGO 2023 staging were detailed in Table 3

### IHC versus NGS concordance

**Fig. 3:**
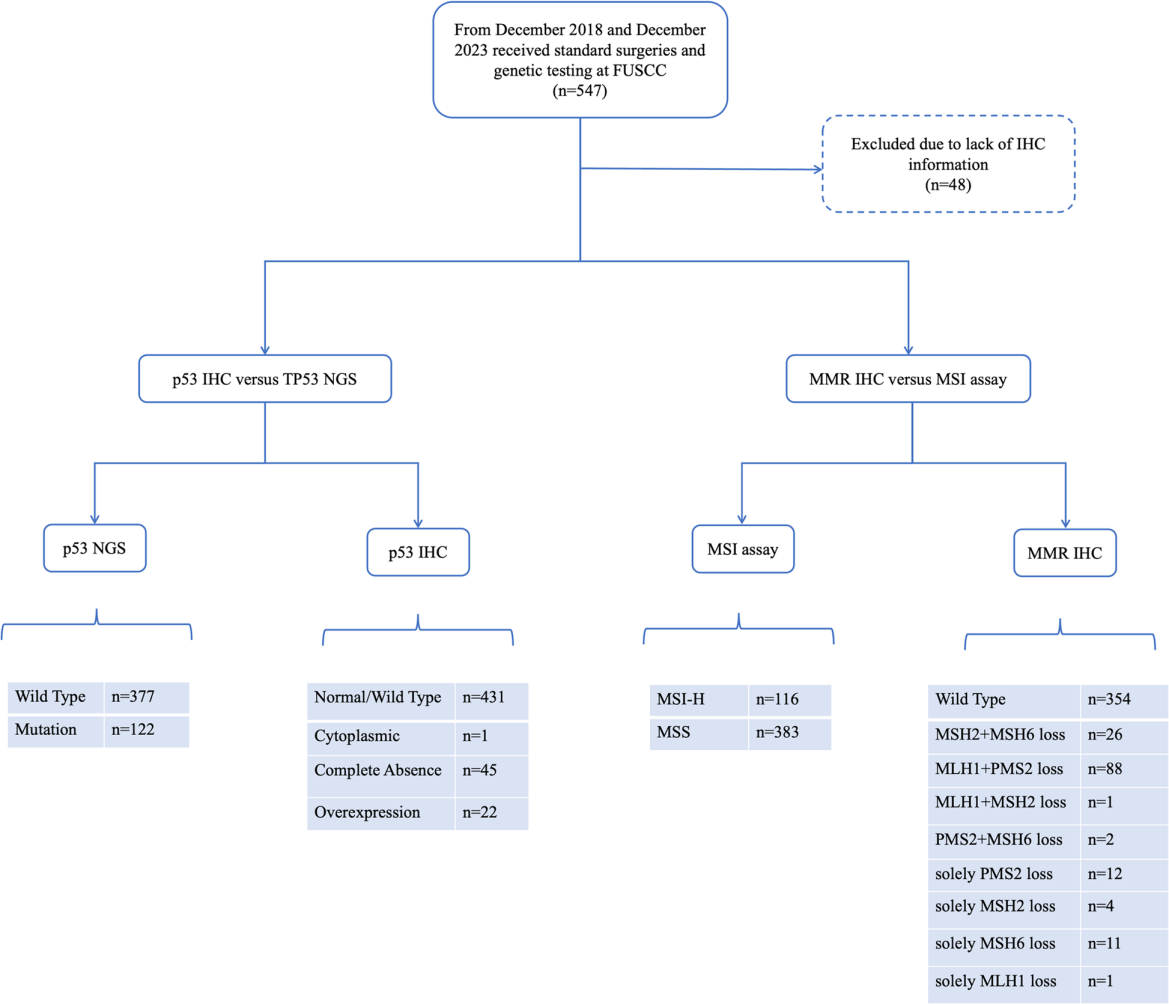
Flow diagram and results of the IHC versus NGS study

Immunohistochemistry (IHC) of p53 protein and MMR protein was detected, which includes p53, MLH1, PMS2, MSH2, and MSH6 protein expression. Forty-eight cases were excluded due to the lack of IHC information. Detailed results of NGS and IHC detection for MSI/MMR and TP53/p53 status are shown in Fig. 3. While taking MMR IHC as a substitution for MSI assay, 38 cases exhibited different results between MMR IHC and MSI assay, with a concordance rate of 92.4%. As for TP53/p53 status, 79 discordant NGS versus IHC cases were observed, representing a discordance rate of 15.8%.

The overall concordance rate was 88.3% when taking the p53 IHC and MMR IHC together into consideration. These discordant situations mentioned above within IHC and NGS result in altered molecular subtypes, especially in the NSMP and p53abn subtypes, as shown in Table 4.

**Table 4:** Alterations in molecular subtypes based on different methods

	NGS ^a^	P53 IHC ^a^	MMR IHC ^a^	P53 & MMR IHC ^a^
NSMP	253	283	230	254
MMRd	111	111	137	139
P53abn	74	44	71	45
*POLE*mut	61	61	61	61

^a^48 samples were excluded due to incomplete immunohistochemical data

^b^next-generation sequencing (NGS)

### Targetable gene alterations

A comprehensive statistical analysis of the NGS panel results was performed (Table 5). PIK3CA and PTEN, pivotal components of the PI3K/AKT pathway, exhibited a high mutation rate of 59% and 81% in all cases, respectively. Especially in the *POLE*mut subgroup, the alteration rates of PIK3CA and PTEN were 88% and 96%, respectively. Genes associated with the homologous recombination repair (HRR) pathway, such as BRCA1/2 and ATM, demonstrated mutation rates of 14%, 26%, and 34% in the MMRd subtype. Additionally, estrogen receptor (ER) and progesterone receptor (PR) exhibited high positivity rates in the NSMP subgroup (by IHC), with positivity percentages of 90% and 88%, respectively. Moreover, in the prognostic analyses, NRAS mutation found as a significant prognostic factor (*p* = 0.039). (Supplementary Fig. 3).

**Table 5:** Gene mutation frequencies in interested pathways

*Targetable alterations*	*POLEmut* *N (%)*	*MMRd* *N (%)*	*NSMP* *N (%)*	*p53abn* *n (%)*	*Chi-square p value *^*a*^
*Total(n)*	69	118	280	80	
*PI3K-AKT pathway*					
PIK3CA	61 (88)	78 (66)	142 (51)	32 (40)	< 0.001
PTEN	66 (96)	108 (92)	224 (80)	45 (56)	< 0.001
KRAS	9 (13)	32 (27)	69 (25)	10 (13)	< 0.05
NRAS	4 (6)	4 (3)	9 (3)	0 (0)	ns
AKT1	5 (7)	6 (5)	29 (10)	5 (6)	ns
*Hormone receptor positivity (based on IHC)*^*b*^					
ER	62 (90)	115 (97)	252 (90)	60 (75)	< 0.001
PR	55 (80)	102 (86)	246 (88)	52 (65)	< 0.001
*Homologous recombination repair*					
BRCA1	27 (39)	17 (14)	9 (3)	7 (9)	< 0.001
BRCA2	49 (71)	31 (26)	28 (10)	8 (10)	< 0.001
ATM	55 (80)	40 (34)	33 (12)	8 (10)	< 0.001
*TGF-*$\beta$* pathway*					
SMAD4	9 (13)	6 (5)	2 (1)	1 (1)	< 0.001
*Receptor tyrosine kinase*					
KIT	30 (43)	11 (9)	3 (1)	1 (1)	< 0.001
PDGFRA	24 (35)	9 (8)	11 (4)	2 (3)	< 0.001

^a^non-significant (ns) refers to *p* > 0.05

^b^Immunohistochemistry (IHC)

## Discussion

In this study, significant shift was observed by restaging with the 2023 FIGO staging system to provide more precise prognostic indications.

The majority of staging alterations occurred in clinically earlier-stage patients (Stages I-II) due to the introduction of molecular subtypes, LVSI status, and aggressive histological types [5]. Patients in the early stages have improved prognostic precision compared with the 2009 system, especially in substages IAm*POLE*mut and IICmp53abn (Supplementary Fig. 2). Multiple studies have confirmed good prognoses of the *POLE*mut subgroup, regardless of postoperative adjuvant therapy [8, 17–20]. In this study, we also verified the excellent prognosis of the IAm*POLE*mut cases, with a 3-year PFS rate of 98.4%. Therefore, de-escalating therapy was suggested by ESGO/ESTRO/ESP guidelines for early-stage *POLE*mut patients [21, 22]. As IICmp53abn patients exhibiting poorer prognoses (3-year PFS rate of 88.3%), regardless of histological type, stage, or grade [23, 24], more advanced therapies were suggested for p53abn EC patients[21]. Moreover, the aggressive histological types were involved in the FIGO 2023 staging system [5]. Previous studies demonstrated the worse prognoses of EC patients with aggressive histology [6, 25]. Our results also figured out that substage IIC patients have significantly worse 3-year PFS rates compared to other stage II diseases with non-aggressive histology type, with 3-year PFS of 72.6% versus 85.6% (IIA) and 100% (IIB), respectively.

Stage shift in stage III diseases was mainly because of the inclusion of the new IA3 category. The introduction of synchronous early-stage uterine and ovarian cancers (SEOs) provides additional refinement in prognosis compared to the 2009 FIGO system. Of note, there were discernible prognostic disparities between IA3 and IIIA1 patients, with a 3-year PFS rate of 100% and 87.5%, respectively. Similarly, Gravbrot et al. also noted significant prognostic distinctions between Stage IIIA and IA3 in their cohort of The National Cancer Database (NCDB) [12]. In the etiology of SEOs, most researchers suggest a complex interplay involving clonality, epigenetics, and the tumor microenvironment, indicating a shared origin associated with a favorable prognosis [26, 27]. Therefore, the postoperative treatment was not necessary for patients with stage IA3.

Besides, in our study, LVSI, aggressive histological subtypes, and lymph node metastases were also identified as significant prognostic factors, which is consistent with previous literature [6–9]. Sentinel lymph node biopsy (SLNB) has been widely used in low-risk EC, and the 37 patients in our study who underwent SLNB demonstrated excellent prognoses. However, there remains controversy regarding the use of SLNB in intermediate-high-risk EC. To address this, a clinical trial (NCT04276532) is currently underway in this patient population. Beyond SLNB, radiomics analysis has emerged as a promising method for predicting nodal metastasis in EC in recent years [28].

ProMisE and TransPORTEC studies have suggested that p53, MLH1, MSH2, MSH6, and PMS2 protein immunostaining status as a substitute for TP53 NGS and microsatellite instability status [3, 29], with an approximate accuracy range of 81.3%-95.6% and 93.3%-98.8% [30–33], respectively. We also employed the IHC as an alternative to molecular testing for economic practicality and clinical accessibility. According to our results, IHC was an acceptable alternative to next-generation NGS in the molecular subtype of EC, especially for mismatch repair protein immunostaining with a concordance rate of 92.4%. However, the concordance rate (84.2%) between IHC and NGS indicated a more cautious decision of interchange of TP53/p53. Discordant results of two techniques testing TP53 status could be explained by tumor heterogeneity, alterations in non-coding regulatory regions, flawed detection of stop-gained mutation, and large-scale deletions or insertions [31, 34]. Some conflicting cases within MMR IHC and MSI assay could be potentially explained by MLH1 promoter methylation, solely MLH1 or MSH6 mutation resulting in protein dysfunction without structural changes, and other undefined situations [33, 35]. In some confusing cases, NGS might be recommended to provide more precise information and avoid misdiagnoses in these patients.

The elevated somatic mutation frequency observed in our study of PIK3CA and PTEN suggests a potential therapeutic strategy targeting the PI3K/AKT/mTOR pathway, and the clinical trials in advanced or recurrent EC showed modest antitumor activity with these targeted therapies. Combined targeted therapy might be an option for these patients [36–39]. In MMRd patients of our study, the highly mutated genes associated with homologous recombination repair pathways suggest potential responsiveness to immune checkpoint inhibition therapy, as previous studies reported [40, 41]. High expression of estrogen and progesterone receptors of NSMP in our study implied the high hormone sensitivity of this subtype. Thereby emerging research has revealed the potential efficacy of anti-estrogen therapy for NSMP patients, such as *Letrozole* and *Anastrozole*, particularly among those at elevated risk of disease recurrence and metastasis [42–44]. As for NRAS mutation, few studies revealed effective therapeutic methods targeting NRAS alone, but targeting MAPK or PI3K pathways may be an indirect but effective therapy (NCT01763164 and NCT01781429) [45–47].

Several limitations should be considered when interpreting the results of our study. Firstly, although our study involved a large sample size, our cohort is confined to a single center which may compromise the power of our findings. Secondly, with the flawed information on lymph node micro-metastasis assessments, the sub-stratification of stages IIIC1 and IIIC2 was unavailable in our study. Thirdly, our results may suffer from a retrospective nature, which may lead to bias inevitably.

In conclusion, in this study, we further explored the clinical applicability and prognostic significance of the 2023 FIGO staging system, and tried to find the potential targeted therapies for different molecular subtypes. Large population-based study is still necessary to validate the accuracy of our findings.

### Supplementary Information

Supplementary Material 1: Supplementary Table 1. Gene list of 46-gene NGS-based panel.

Supplementary Material 2: Supplementary Figure 1. The inclusion and exclusion criteria.

Supplementary Material 3: Supplementary Figure 2. Kaplan-Meier survival analyses of different variables.

Supplementary Material 4: Supplementary Figure 3. Kaplan-Meier survival analyses of genes in key pathways.

## Data Availability

The datasets used and/or analysed during the current study are available from the corresponding author on reasonable request.
