# Supplementary figures and images for: Clinical application of FIGO 2023 staging system of endometrial cancer in a Chinese cohort
Source: BMC Cancer. 2024 Jul 18;24:862. doi: 10.1186/s12885-024-12633-8 (PMC11264810; doi:10.1186/s12885-024-12633-8)

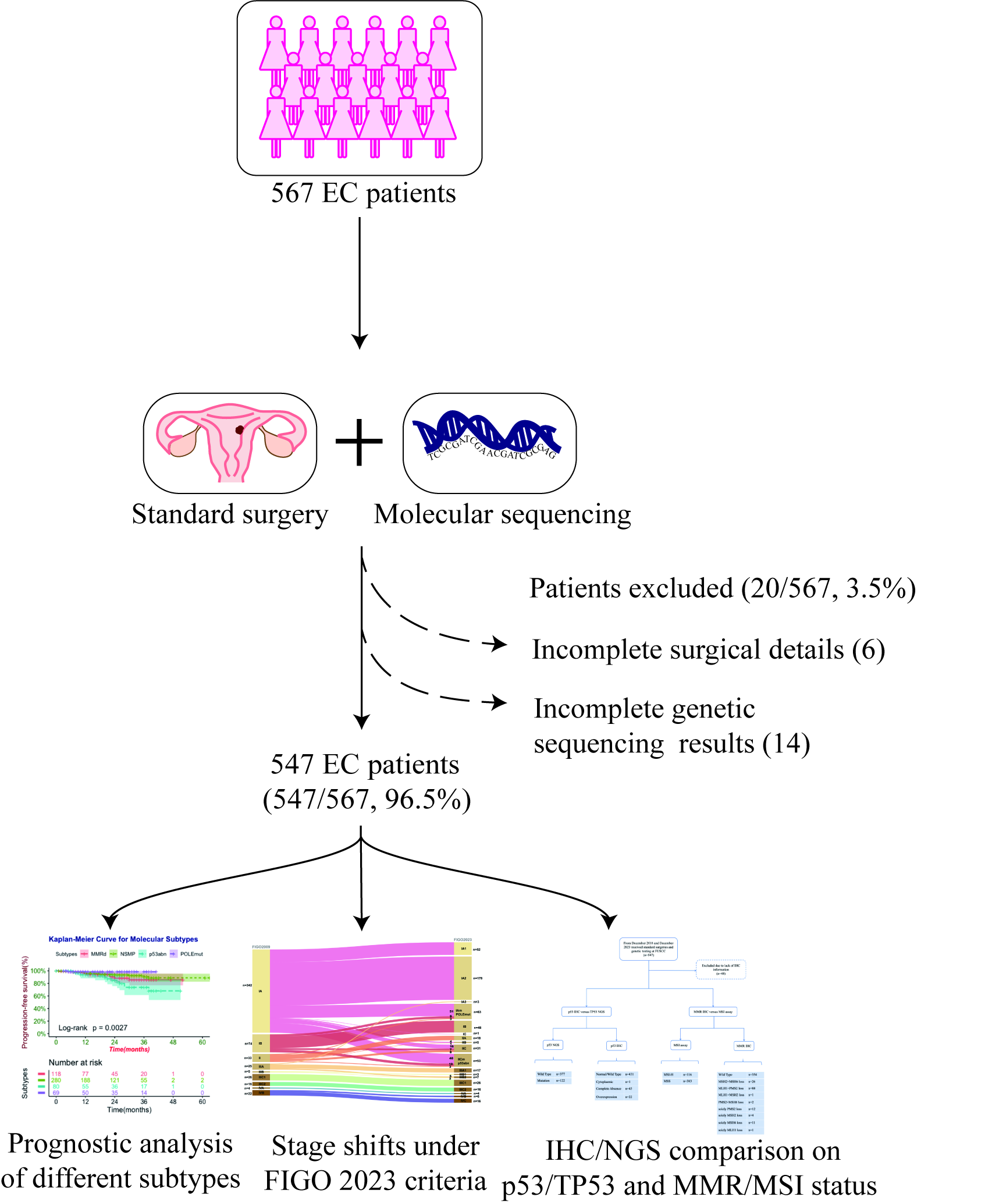

Supplement: Supplementary file 2 — Supplementary Material 2: Supplementary Figure 1. The inclusion and exclusion criteria. [file 12885_2024_12633_MOESM2_ESM.tif]

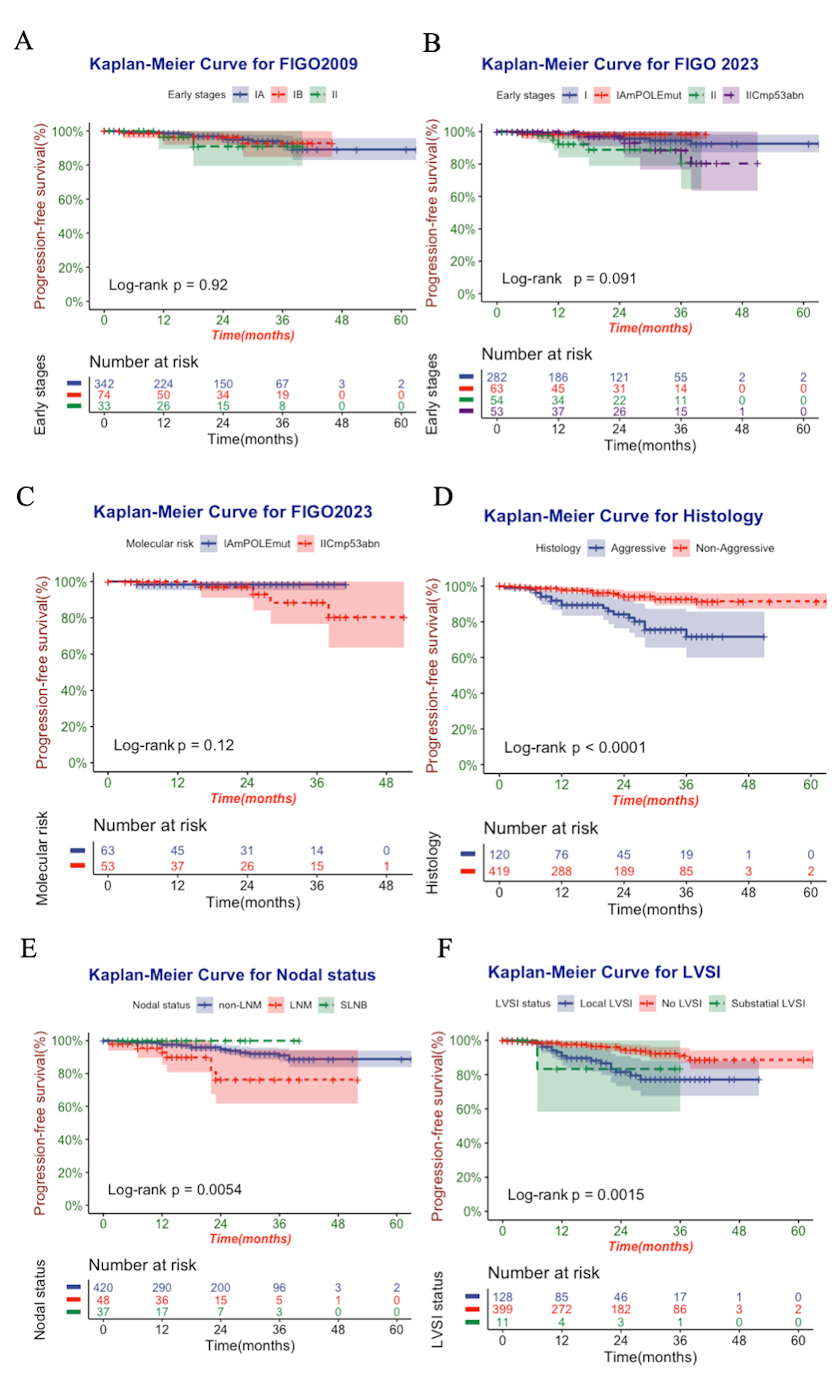

Supplement: Supplementary file 3 — Supplementary Material 3: Supplementary Figure 2. Kaplan-Meier survival analyses of different variables. [file 12885_2024_12633_MOESM3_ESM.tiff]
